# Designing and Testing a Treatment Adherence Model Based on the Roy Adaptation Model in Patients With Heart Failure: Protocol for a Mixed Methods Study

**DOI:** 10.2196/13317

**Published:** 2019-07-26

**Authors:** Shabnam Shariatpanahi, Mansoureh Ashghali Farahani, Forough Rafii, Maryam Rassouli, Amir Kavousi

**Affiliations:** 1 Nursing Care Research Center School of Nursing and Midwifery Iran University of Medical Sciences Tehran Islamic Republic of Iran; 2 Cancer Research Center, Shahid Beheshti University of Medical Sciences Tehran Islamic Republic of Iran; 3 Workplace Health Promotion Research Center and Department of Epidemiology, School of Public Health and Safety, Shahid Beheshti University of Medical Sciences Tehran Islamic Republic of Iran

**Keywords:** adaptation, treatment adherence and compliance, heart failure

## Abstract

**Background:**

Adherence to treatment is an important factor to decrease repeated and costly hospitalization owing to heart failure (HF). The explanation and prediction of medication adherence and other lifestyle recommendations in chronic diseases, including HF, are complex. Theories lead to a better understanding of complex situations as well as the process of changing behavior and explain the reasons for the existence of a problem.

**Objective:**

The aim of this study is to report a protocol for a mixed methods study setting out to investigate the empirical validity of the Roy Adaptation Model as a conceptual framework for explaining and predicting adherence to treatment in patients with HF in Iran.

**Methods:**

This mixed methods study consists of an exploratory sequential design to be conducted in 2 phases. The first phase involves identifying the factors associated with treatment adherence in patients with HF through content analysis of the literature and elucidating the perception of participants in the context of Iranian health care where the model of adherence to treatment is designed based on the Roy Adaptation Model. The second phase addresses the interrelationships among variables in the model through a descriptive study using structural equation modeling. Finally, following the summarization and separate interpretation of the qualitative findings and quantitative results, a decision is made about the extent to and ways in which the results of the quantitative stage can be generalized or tested for the qualitative findings.

**Results:**

Content analysis of the literature in part 1 of the first phase was completed in 2017. Collection and analysis of qualitative data in part 2 of the first phase will be completed soon. The results are expected to be submitted for publication in 2019. Then, the second phase—the quantitative study—will be conducted.

**Conclusions:**

The results of this study will provide valuable information about the empirical validity of the Roy Adaptation Model as a conceptual framework for explaining and predicting adherence to treatment in patients with HF, which, to date, have received little attention. The results can be used as a guide for nursing practice and care provision to patients with HF and also to design and implement effective interventions to improve treatment adherence in these patients.

**International Registered Report Identifier (IRRID):**

DERR1-10.2196/13317

## Introduction

### Background

Heart failure (HF) is a highly prevalent chronic disease that is considered a global epidemic [1]. A total of 5.1 million people in the United States suffer from this disease, the prevalence of which is anticipated to reach 46% by 2030 [2]. The prevalence of HF in Iran was reported as 8% in 2014 [3]. Despite the significant progress in the treatment of this disease, the mortality and readmission rates associated with this condition have remained high, with a 5-year survival rate of 50% [2] and with rehospitalizations common in HF patients; 83% of patients were hospitalized at least once and 43% hospitalized at least 4 times. These values did not differ in HF with reduced Ejection Fraction (EF) versus preserved EF [2]. These rates lead to increased costs not only for the patients and their family but also for the society and the health care system. Costly readmissions, mortality, morbidity, and progress of the disease can be reduced in these patients through adherence to the treatment regimen [4-8].

Adherence to treatment has been defined as a range of medication behaviors and lifestyle choices that are consistent with the regimen recommended by health care providers [9]. The rate of adherence reported in HF studies has varied within 7% to 90%, depending on differences in methodological approaches such as the type of definition and measurement method of adherence and the criteria for selecting patients in each study [10]. There is little information about the rate of adherence with the treatment regimen in HF patients in Iran. In one study [11], 60.3% of patients and in another study [12], 71.4% of patients had poor self-reported medication adherence. Adherence to treatment in HF is a multidimensional phenomenon influenced by the interplay of several factors, such as socioeconomic factors (age, gender, race, and lack of access to transportation), health care system–related factors (inadequate education of health care providers in chronic diseases, shortened time of visits by the physician, and lack of insurance support), patient-related factors (forgetfulness, improvement or reduction of symptoms, and knowledge deficiency), condition-related factors (severity of illness and depression), and therapy-related factors (side effects and the number of medications taken during the day) [9,13-15]. The explanation and prediction of medication adherence and other lifestyle recommendations in chronic diseases, including HF, are complex and should be understood before intervention [15]. Theories contribute to a better understanding of the complex situations as well as the process of changing behavior and explain the reasons for the existence of a problem [16]. Indeed, theories can provide a framework for explaining or predicting the likelihood of events and facilitate an understanding of various components and behavioral dynamics [17]. There are several social cognitive theories for explaining, predicting, and modifying adherence behavior with treatment regimen [18]. Meanwhile, application of nursing theories as a conceptual framework in research is essential for the development of nursing knowledge [19,20]. The use of conceptual models to guide nursing research seems complicated because of their abstract concepts. The use of a conceptual model for conducting nursing research requires construction of a conceptual-theoretical-empirical(C-T-E) structure. In this structure, the component C is a conceptual model containing a set of general and abstract concepts and propositions that are used as the basis for research. The component T refers to the theory extracted from the conceptual model, which can be translated into empirical indicators (E) and tested further [21]. Note that the C-T-E structure for nursing is important, as it leads to the structure of the body of nursing knowledge and provides a reference framework for researcher nurses or nurses who take care of patients with a chronic disease on how to observe and interpret the phenomena of interest in the discipline [22]. The relationship between theory, research, and clinical practice is essential for the continuous development of nursing as a profession and science. Ideally, clinical practice should be based on the theory validated by research [23].

The Roy Adaptation Model in nursing is one of the most widely used conceptual frameworks for guiding research, nursing practice, and training. Furthermore, given its scope, it can be used as a conceptual model to generate and test nursing theories [24,25] because adherence to treatment is an adaptive behavior that is essential for coping with chronic diseases [26]. As a conceptual model of adaptation, the Roy Adaptation Model appears capable of explaining adherence behaviors in these patients. The Roy Adaptation Model is an effective guide for nurses’ practice across all settings [27] including HF [28]. According to the Roy Adaptation Model, a person is a bio-psycho-social being who is in constant interaction with the physical and social environment and uses adaptive strategies to maintain balance [29]. On the basis of this conceptual framework, the purpose of nursing is to improve adaptation [26] and enhance adherence in patients. Adherence behaviors help people achieve physiological, psychological, and social adaptation [30].

Accordingly, this study was conducted to determine the empirical validity of the Roy Adaptation Model as a conceptual model for explaining and predicting treatment adherence in patients with HF in Iran using a mixed-methods research. Theory testing studies have been designed to examine middle-range theories through the process of deductive reasoning. To this end, concepts and the statement of the middle-range theory were weighed up against those of the conceptual model, and the middle-range theory was then tested using research techniques and empirical indicators [20].

### The Roy Adaptation Model

The Roy Adaptation Model has major concepts of nursing theory including health, person, nurse, and environment. In this conceptual model, the person has been viewed in a holistic manner. The main concept in this model is adaptation. Adaptation is considered as a process and an outcome with 3 integrated, compromised, and compensatory levels. The person as an adaptive system uses 2 mechanisms of coping (the cognator and the regulator) to cope with the changing world. The function of the subsystems is to maintain integrated adaptation. The pooled effect of the focal, contextual, and residual stimuli establishes a person’s adaptation level. The focal stimuli are internal or external stimuli that are immediately present in the consciousness of the individual. On the contrary, contextual stimuli are other factors emerging in situations that contribute to the focal stimulus effect. Finally, the residual stimuli are environmental factors in or out of the person, the effects of which are not clear in the current situation. Adaptation levels are present in people’s behavior, where the behaviors can be observed in 4 adaptive modes (physiological, self-concept, interdependence, and role function).

Behavior in the physiological mode reflects the physiological processes of cells, tissues, organs, and body systems. The physiological mode has 5 basic physiological needs (activity and rest, nutrition, elimination, oxygenation, and protection) and 4 regulatory processes (senses, fluids - electrolytes and base acid balance, neurological function, and endocrine function). Self-concept is a set of feelings and beliefs about oneself that are shaped by the inner perception and understanding of the reactions of others. The components of this mode include physical self (such as physical sensation and body image) and personal self (self-consistency, self-ideal, and moral-ethical-spiritual self). The role function is a role-related behavior aiming at achieving social integrity. The role is a set of expectations about how a person functions in the society in relations to others. The interdependence mode includes behaviors related to interdependent relations of individuals. This mode focuses on interactions related to giving and receiving love, respect, and value. The basic need for this mode is relational integrity [31].

## Methods

This paper describes the protocol of a mixed-methods study with a sequential exploratory design on the adherence to treatment regimen in patients with HF in Iran. This model starts with data collection and analysis of a qualitative phase, which is then followed by quantitative data collection and analysis [32]. Steps of implementing the study with a sequential exploratory approach adapted from Creswell and Clark are shown in Textbox 1 [33].

**Steps of implementing the study with a sequential exploratory approach.**

**First phase**

- **Stage 1**
  Designing the qualitative study
  - Objective: Re-designing the model of adherence to treatment in patients with heart failure based on the Roy Adaptation Model
  - Type of research: Directed qualitative content analysis
  Implementation of the qualitative study
  - Content analysis of relevant literature in part 1 based on the modes and stimuli of the Roy adaptation model and re-designing of the model of adherence to treatment in patients with heart failure based on the Roy adaptation model
  - Interviewing patients with heart failure and their caregivers as well as analyzing the data based on the modes and stimuli of the Roy adaptation model in part 2
  - Presenting the final model of adherence to therapeutic regimen in patients with heart failure

- **Stage 2**
  Strategies of relating the primary qualitative study to the secondary quantitative study
  Using qualitative findings to:
  - Determine research hypotheses for the quantitative phase
  - Designing a study for the quantitative phase

**Second phase**

- **Stage 3**
  Objective: Testing the model by determining the interrelationships among variables
  Type of research: Descriptive-correlational
  - Determining sample size based on the number of factors in the model
  - Stratified random sampling with proportional allocation considering the mean number of patients with heart failure in educational health care centers of the selected university of medical sciences
  - Analyzing quantitative data through structural equation modeling

- **Stage 4**
  Interpretation of connected results
  - Summarizing and interpreting the qualitative results
  - Summarizing and interpreting the quantitative results
  - Discussing how and to what extent quantitative results match qualitative results

**Table 1:** Search strategy.

Database	Search terms
PubMed	(“failure” OR “heart failure” OR “myocardial failure” OR “CHF” OR “HF” OR “congestive heart”) AND (“patient concordance” OR “patient cooperation” OR “patient adherence” OR “patient compliance” OR “treatment refusal” OR “patient dropouts” OR “self-care maintenance”)
Scopus	(ALL(“failure”) OR ALL(“heart failure”) OR ALL(“myocardial failure”) OR ALL(“CHF”) OR ALL(“HF”) OR ALL(“congestive heart”)) AND (ALL(“patient concordance”) OR ALL(“patient cooperation”) OR ALL(“patient adherence”) OR ALL(“patient compliance”) OR ALL(“treatment refusal”) OR ALL(“patient dropouts”) OR ALL(“self-care maintenance”))
Web of Science	(TS=(“failure”) OR TS=(“heart failure”) OR TS=(“myocardial failure”) OR TS=(“CHF”) OR TS=(“HF”) OR TS=(“congestive heart”)) AND (TS=(“patient concordance”) OR TS=(“patient cooperation”) OR TS=(“patient adherence”) OR TS=(“patient compliance”) OR TS=(“treatment refusal”) OR TS=(“patient dropouts”) OR TS=(“self-care maintenance”))

### The First Phase: Qualitative Study

This part of the study is performed in 2 parts: in part 1, factors associated with treatment adherence in patients with HF corresponding to the focal and contextual stimuli of the Roy Adaptation Model affecting the physiologic, self-concept, role function, and interdependence modes are identified and categorized according to a content analysis of the literature. To this end, a search is carried out on articles published between 1970 and 2017 in a number of databases, including Scopus, PubMed, and Web of science (Table 1). In addition, research reports and dissertations are also included in the search to have a comprehensive search. A manual search is also performed in the list of resources extracted from the studies. This literature review examines only studies in which the participants are adult patients with HF (aged 18 years and older) or one of its population subgroups with class I to IV HF according to the New York Heart Association (NYHA) Classification or patients with reduced ejection fraction(HFrEF), preserved ejection fraction (HFpEF), and mid-range ejection fraction (HFmrEF). In addition, studies that have investigated one or more components of adherence to treatment (medication adherence and nonpharmacological (lifestyle) recommendations), have examined adherence as the focal outcome, have reported their results in qualitative and quantitative forms, are in English or Persian, and give access to their full text are considered as eligible. On the contrary, editorials, letters to editors, and summaries of conferences are excluded. Taking into account these inclusion and exclusion criteria, the title and abstract of the articles retrieved through the initial search are examined and articles relevant to the objectives of this content analysis of the literature are then selected. Next, the full text of the eligible articles is reviewed and analyzed using qualitative content analysis with an approach guided by the Roy Adaptation Model modes as well as focal and contextual stimuli. This step also involves identifying and categorizing the features of adaptive and nonadaptive behaviors in each mode. Next, the initial model of treatment adherence in patients with HF is then redesigned based on the modes and stimuli proposed in the Roy Adaptation Model.

In part 2, to redevelop a Roy Adaptation Model–based model of treatment adherence in patients with HF, the factors associated with treatment adherence are examined based on the context of Iranian cultural from the perspective of the patients, their families, and health care providers using qualitative content analysis. This study used directional content analysis, which is a method used when there is an initial theory or a basic research about the phenomenon in question which may be incomplete or can be used in future research. This approach aims to validate or develop the conceptual framework of a theory, where the existing theory or initial research can serve as a guide for investigating the relationship between the codes [34]. In this study, after redesigning and redeveloping the Roy Adaptation Model–based model of treatment adherence in the current Iranian cultural context, the research hypotheses are determined and the quantitative stage of the study is designed.

#### Study Population and Setting

As in a qualitative study, the study setting is where the phenomenon actually occurs [35], this study is conducted in 6 Referral Cardiovascular Centers affiliated with universities of medical sciences in Tehran with a cardiology unit, cardiac care unit, and heart clinic alongside other places where access to participants is possible (such as cardiologists’ offices). This part of the study focuses on patients with HF (NYHA class I-IV or patients with HFrEF, HFpEF, and HFmrEF) coupled with their caregivers and health care providers. The patients’ caregivers and health care providers are selected because they are involved in providing care to adults with chronic conditions.

#### Sample Size and Sampling Method

The sample size is unknown in this part of the study, and sampling continues until the saturation of data, that is, sampling to the point at which no new information is obtained and redundancy happens [35]. After reaching repeated information, interviews are conducted with more participants to ensure no new data are obtained.

The sample was selected using purposive sampling (the researcher purposely selects participants who can act as a rich source of information and can take part in the study and help the researcher better understand the factors related to and affecting treatment adherence—using the strategy of maximal variation sampling). This means that participants are purposefully selected to be diverse in age, socioeconomic situation (level of education, living arrangement, marital status, employment status, and gender), duration of the disease, and illness severity according to class I to IV NYHA Classification (with higher class indicating more severe symptoms).

The patient sample included persons with a primary diagnosis of HF for more than 3 months meeting the following criteria: at least 18 years of age and NYHA Classification I to IV or patients with HFrEF, HFpEF, and HFmrEF. The exclusion criteria included patients suffering from terminal diseases, cognitive impairments, obvious psychiatric disorders with limited capacity to engage in self-care, and candidacy for heart transplantation in the 6 upcoming months. The health care provider sample included cardiologists and nurses providing direct care to adult patients with HF with at least one year of work experience in this field. The patient caregivers’ sample included an adult family member, such as a spouse sharing residence with the patient with HF and responsible for taking care of the patient or acting as the patient’s caregiver at least twice per week.

#### Data Collection

The main method for data collection in this part of the research is a semistructured interview using open questions and field notes, but other data collection methods such as observation and existing documents will also be used. Using the directional content analysis, the researcher begins the interviews with general open-ended questions and tries to have the minimum interference in the process of the interviews. The researcher also uses directional questions according to the Roy Adaptation Model and directs the interview such that it covers the study objectives using guiding questions; for example:

1. Please explain about your heart disease.
2. What do you do to take care of yourself concerning your illness? Why?
3. How do you feel about your treatment regimen? Talk about feelings you experience physically or emotionally after taking medication, dietary restriction, or physical activity and other treatment related issues.
4. Have you ever had a chance to forget your diet (eg, you forget to take one or more medication doses)? What was the reason and when this happened to you?
5. Are there any situations that make it easier for you to follow medical treatment regimen (eg, taking medicine, diet, and physical activity)?
6. What is the most difficult aspect of adherence to treatment regimen (eg, the most difficult aspect of medication use)?

Probing questions are then asked for clarifying the subject based on the information provided by the participants. In-depth questions, such as *Please elaborate*, *What do you mean?,* and *Can you give an example to help me understand better?* are also asked according to the given responses. During the interview, the researcher makes a detailed note of the nonverbal data, such as tone of voice, facial expressions, and state along with their time and place. The duration of the interviews will be between 45 and 90 min depending on participants’ ability to answer the questions and all will be audio-recorded. After each interview, the content of the interviews is transcribed verbatim after several times of listening in the first possible instance and is further analyzed and coded as it is being collected using directional content analysis. The interviews continue until data saturation, when no further new data or codes are obtained. After reaching repeated information, interviews are conducted with more participants to ensure no new data are obtained.

#### Data Analysis

MAX Qualitative Data Analysis software for qualitative data analysis (version 10) was used for data management. Advantages of this software include the ability to handle a large amount of information, saving time, the flexibility, and the possibility of performing more complex analyses. In the qualitative part, data are analyzed as they are being collected using directional content analysis. Directional content analysis has 3 phases including preparation, organizing, and resulting [36]. In the preparation phase, the written material (the text of transcribed interviews and observations) is read several times for data immersion. In the organizing phase, an unconstrained categorization matrix is developed based on the concepts of the Roy Adaptation Model. Then, the researcher reviews the text line-by-line and encodes it. The initial codes are then reviewed several times and then categorized according to the differences and similarities between them. The categorized codes are assigned to larger categories as subthemes (these categories are derived from the concepts of the initial redesigned model based on the Roy Adaptation Model). If the categorized codes are not suitable as a subtheme of the initial model, they are categorized as separate subthemes. Eventually, these categories are categorized as a main theme of the initial model, and the initial model is thus developed according to participants’ perceptions of treatment adherence.

#### Data Rigor

The quality of the data is assessed using Lincoln and Guba’s criteria [37]. In this study, the rigor of the data is ensured by member check. The results of the study are presented to some of the participants, and their review ensures consistency and further adds to the credibility of the data. Peer debriefing is another method used to confirm the credibility of data. To this end, the interview texts and the extracted codes and categories are reviewed and assessed not only by advisors and supervisors but also by a faculty member with expertise in qualitative studies to determine the accuracy of the encoding process. Prolonged engagement is another measure taken to enhance the credibility of the study findings. This study also seeks to realize the confirmability of the results by clearly documenting every stage of the study. In addition, the transferability of the results to similar settings becomes possible for other researchers by seeking to cover a wide range of participants in terms of age, occupation, marital status, severity of the disease, duration of the disease, and education as well as by describing the study context and participants.

### The Second Phase: Quantitative Study

The model is tested to investigate the relationship between the hypotheses or variables. In this process, all the variables of the model are measured to determine the path between the concepts and develop the conceptual model. In this part of the study, the model is validated through a descriptive study using valid and reliable tools related to the model variables and by applying Structural Equations Modeling. Eventually, the final model developed based on the cultural and socioeconomic characteristics of the Iranian society is presented.

#### Study Population and Setting

The study setting in this part of the study is similar to part 2 of the first phase including 6 Referral Cardiovascular Centers affiliated with universities of medical sciences in Tehran. The study population consists of all the patients with HF (NYHA class I-IV or patients with HFrEF, HFpEF, and HFmrEF) presenting to these centers.

#### Sample Size and Sampling Method

Determining the minimum sample size required for collecting data related to the structural equation modeling is very important. There is no specific rule for choosing the right sample size, which makes this step more difficult. For instance, some researchers recommend 10 samples per parameter or variable, whereas some references consider 200 as the minimum sample size required for factor analysis and structural equation modeling [38]. This study selects a sample size of 10 per variable.

In the sampling process, proportional stratified random sampling is conducted in the 6 Referral Cardiovascular Centers based on the mean number of patients with HF in each Center. In the next step, the same sampling method is used to choose patients based on their NYHA function classes (I-IV) to ensure the enrollment of equal number of individuals with different classes of HF.

The inclusion and exclusion criteria in this part of the study are similar to characteristics of patients with HF in part 2 of the first phase.

#### Scales and Data Collection

Treatment adherence has been defined as an adaptive behavior which indicates people’s cost-benefit perceptions of treatment manifested as integrated (100% adherence), compensatory (relative or assisted adherence), and compromised (nonadherence) [39]. This study considers treatment adherence as the latent variable which reflects the adaptation system’s general response to environmental stimuli (ie, factors related to treatment adherence) and is manifested in the 4 modes of bio-psycho-social response. The data collection tool used for testing the model is, therefore, a demographic form where the instruments used for measuring the concepts of the treatment adherence model are developed according to the Roy Adaptation Model obtained from the qualitative part.

#### Data Rigor

To ensure the rigor of the data in the quantitative part, valid and reliable tools are used to measure the concepts and variables of the Roy Adaptation Model–based treatment adherence model. To assess the validity of the questionnaires, the face and content validity methods are used. For assessing the face validity of the Persian version of the scales, they are distributed among 10 patients with HF to comment on the clarity and comprehensibility of the items, whereby their corrective views and suggestions are collected and implemented, and the final version is then drafted and approved by faculty members.

To assess the content validity, the questionnaires are distributed among 15 faculty members of schools of nursing and midwifery in Iran who are experts in the field, whose views are collected. After getting the qualitative feedback of the faculty members and making the necessary corrections, content validity is assessed quantitatively using the Content Validity Index [35]. The reliability of the questionnaire is confirmed using the Cronbach alpha coefficient.

Information bias, nonresponse bias, and selection bias are among the potential sources of bias in this phase of study. To reduce information bias, the questionnaires would be completed through face-to-face interviews by interviewers who are unaware of the hypothesis under consideration. To reduce nonresponse bias, a financial reward is offered to participants. Finally, proportional stratified random sampling is used to reduce selection bias.

#### Data Analysis

In this part, structural equation modeling is used to test the model in linear structural relations. A statistical software package is used to test the validity of assumed models and to illustrate the relationship between variables [40]. Structural equation modeling is a combination of confirmatory factors and path analysis, taking into account the latent variables that cannot be directly measured. Structural equation modeling is used in various cases, for instance, it is applicable when there are latent variables with each having several indices (confirmatory factor analysis) or when the assumed paths between different variables need testing, or when there is a comprehensive approach based on a theoretical model [41,42].

### Ethics Approval and Consent to Participate

This research project is approved under the ethics code IR.IUMS.REC.1395.9221199205 by Iran University of Medical Sciences. Before conducting the study, its objectives and methods are explained to the candidates who accept the researcher’s invitation to take part. Informed written consent is then obtained from them for participation in the study (including a consent for recording the interviews and completing the questionnaires). The study subjects are ensured of their right to withdraw from the study at any time and their anonymity in all the stages of the publication of the results, and they are later presented with the results of the research if they so wish.

## Results

### Interpretation of Interrelated Results

The interpretation of interrelated results is referred to as *drawing conclusions* or *drawing inferences*. Although inferences can be drawn after each stage of the study, meta-inferences are drawn at the end of the study, which include a larger interpretation in the Conclusions or Discussion sections of the study. In an exploratory study, meta-inferences determine whether the quantitative part of the follow-up provides a more generalized understanding of the study subject compared with the qualitative data alone. This interpretation ends in responding to a combination of questions [32]. In this part of the study, following the summarization and separate interpretation of the qualitative findings and quantitative results, a decision is made about the extent to and ways in which the results of the quantitative stage can be generalized or tested for the qualitative findings. Ultimately, the empirical validity of the Roy Adaptation Model as a conceptual model is determined for predicting treatment adherence in patients with HF.

### Current Status of the Study

Content analysis of the literature in part 1, the first phase, was completed in 2017. Collection and analysis of qualitative data in part 2, the first phase, will be completed soon. The results are expected to be submitted for publication in 2019. Then, the second phase—the quantitative study—will also be conducted.

## Discussion

### Summary

There are different theories that provide a framework for the study of nursing requirements and outcomes in different settings as well as for patients in different times of their life. The Roy Adaptation Model is one of the most useful conceptual models in nursing which can be used in research, training, clinical practice, and health care system management [43]. It provides an effective framework for assessing people’s adaptation to various environmental stimuli regardless of their age and disease. As a conceptual model of adaptation, the Roy Adaptation Model is applicable to and flexible for different approaches, projects, objectives, settings, and age groups [24]. Nursing knowledge can be developed through research based on Roy Adaptation Model [44]. Fawcett quoted from Roy and Robbert that the theory of person as an adaptive system should be developed and tested by a systematic program of research [45]. To the researchers’ knowledge, this study is the first to determine the empirical validity of the Roy Adaptation Model as a conceptual model for predicting treatment adherence in patients with HF in Iran through a mixed-methods study.

The results of this research will help identify the factors associated with treatment adherence and better understand the perceptions of patients with HF about treatment adherence within the Iranian cultural and socioeconomic context. These results also provide an insight into the perceptions of the families of patients with HF as well as health care providers about treatment adherence and its related factors in HF. By obtaining a more profound understanding of the perceptions of patients with HF and their caregivers as well as health care providers and by conducting studies supported by nursing theories as the road map, researchers will be able to design efficient interventions to improve treatment adherence in these patients and assess them within this conceptual framework. This study can help develop a checklist for assessing the variables responsible for nonadherence behaviors in this group of patients derived from the Roy Adaptation Model stimuli (focal, contextual, and residual) and identify the practical priorities for improving adherence to treatment regimen in this group.

### Limitations

Our study has some limitations. First, the aim of this study is to provide a treatment adherence model for patients with different severities of illness (NYHA class I-IV or patients with HFrEF, HFpEF, and HFmrEF). So, one of the potential limitations of the study may be the inability of patients in class III-IV to answer a question. Second, adherence to the treatment regimen is influenced by the health care system, so some of the possible themes associated with these factors may be underreported. Finally, the withdrawal of patients with cognitive impairment and end-of-life diseases from the study leads to exclusion bias, complicating the generalizability of the results for this group of patients.

In the quantitative phase, reliable and valid tools are needed to test the interrelationships between variables. As the concepts of the treatment adherence model based on the Roy Adaptation Model at this stage are not clear, it is difficult to determine the tools required for testing the model in the quantitative phase of the study. On the other hand, it may be necessary designing a new tools or translating existing tools and cultural adaptation of them, which is one of the limitations of this study as the exploratory sequential mixed-methods research design.

## Abbreviations

C-T-E: conceptual-theoretical-empirical
EF: Ejection Fraction
HF: heart failure
HFmrEF: HF with mid-range ejection fraction
HFpEF: HF with preserved ejection fraction
HFrEF: HF with reduced ejection fraction
NYHA Classification: New York Heart Association Classification
